# Strengths and limitations of a tool for monitoring and evaluating First Peoples’ health promotion from an ecological perspective

**DOI:** 10.1186/s12889-015-2550-3

**Published:** 2015-12-08

**Authors:** Kevin Rowley, Joyce Doyle, Leah Johnston, Rachel Reilly, Leisa McCarthy, Mayatili Marika, Therese Riley, Petah Atkinson, Bradley Firebrace, Julie Calleja, Margaret Cargo

**Affiliations:** Onemda VicHealth Koori Health Group, Centre for Health Equity, Melbourne School of Population & Global Health, The University of Melbourne, Melbourne, VIC 3010 Australia; Wardliparringa Aboriginal Research Unit, South Australian Health and Medical Research Institute, Adelaide, SA 5000 Australia; Menzies School of Health Research, Alice Springs, NT 0870 Australia; Centre for Excellence in Intervention and Prevention Science, Carlton, VIC 3053 Australia; Viney Morgan Aboriginal Medical Service, Barmah, VIC 3639 Australia; School of Population Health, University of South Australia, Adelaide, SA 5000 Australia

**Keywords:** Health promotion, First Peoples, Aboriginal and Torres Strait Islander, Evaluation, Determinants of health, Ecological, Systems

## Abstract

**Background:**

An ecological approach to health and health promotion targets individuals and the environmental determinants of their health as a means of more effectively influencing health outcomes. The approach has potential value as a means to more accurately capture the holistic nature of Australian First Peoples’ health programs and the way in which they seek to influence environmental, including social, determinants of health.

**Methods:**

We report several case studies of applying an ecological approach to health program evaluation using a tool developed for application to mainstream public health programs in North America – Richard’s ecological coding procedure.

**Results:**

We find the ecological approach in general, and the Richard procedure specifically, to have potential for broader use as an approach to reporting and evaluation of health promotion programs. However, our experience applying this tool in academic and community-based program evaluation contexts, conducted in collaboration with First Peoples of Australia, suggests that it would benefit from cultural adaptations that would bring the ecological coding procedure in greater alignment with the worldviews of First Peoples and better identify the aims and strategies of local health promotion programs.

**Conclusions:**

Establishing the cultural validity of the ecological coding procedure is necessary to adequately capture the underlying program activities of community-based health promotion programs designed to benefit First Peoples, and its collaborative implementation with First Peoples supports a human rights approach to health program evaluation.

## Background

Since the 1980s the adoption of ecological frameworks for targeting and evaluating health promotion interventions has gained momentum as approaches for planning and assessing complex interventions, with multiple strategies at individual and environmental levels, have been developed [1–4]. Discussions of this approach in general have focussed on its (very strong) rationale for influencing health outcomes through targeting determinants at both individual and multiple environmental levels (e.g. [5, 6]). However, we are not aware of published studies examining the quantitative relationship between the degree to which programs integrate an ecological approach and the magnitude of the associated changes in health outcomes, although Stokols’ later review [2] cites three examples of successful application of the ecological approach in U.S. smoking and road safety programs. It is also clear that smoking prevalence in Australia has responded to multi-level intervention at a national level [7].

An ecological approach thus allows assessment of how health programs address the ‘social determinants of health’. Social determinants are conventionally taken to mean employment, income, education, housing and other indicators related to Western cultural norms. From a First Peoples’ perspective, social determinants can include a much broader array of influences including cultural genocide and survival, Land, family support and connection, relationships with mainstream/dominant society and First Peoples’control over their own health, and it also encompasses issues of Human Rights and health equity [8–10]. Hence the application of such an approach (or any other approach) must be at the direction of First Peoples in order to meet standards of rigour in data collection and interpretation and to meet human rights obligations regarding First Peoples’ access to information about their own health [11]. In this paper we have used a definition of social determinants that encompasses this breadth of influences identified by First Peoples, and incorporated the associated processes of leadership by and collaboration with First Peoples.

Internationally, the evaluation of health programs has been problematic in the absence of local community input and control over what is monitored and how, what data mean and what constitutes ‘success’, and the need to overcome a dominant postpositivist approach that ignores important contextual factors such as social determinants [12]. Likewise in Australia, there is a gap in knowledge on how to evaluate the extent to which health promotion activities address determinants of First Peoples’ health. Ecological theory recognises the influence of the social and physical environment on wellbeing, and an ecological approach to health promotion is more aligned with the holistic, ‘whole of community’ approach favoured by Aboriginal Community Controlled Organisations (ACCOs) and with current frameworks and policies [13, 14]. As such, it has the potential to at least partly meet the identified need to develop methods that come closer to measuring the impact of interventions, which are often complex and multifaceted [15]. Ecological theory provides a lens for understanding and describing the multi-level nature of communities, with interactions between the social, physical and policy systems in which people live. It is in this context that we have investigated methods for capturing the purpose and design of health programs which seek to address First Peoples’ health in Australia.

In the Aboriginal and Torres Strait Islander health research literature an ecological approach appears explicitly in an increasing number of reports, including: a longitudinal evaluation of health initiatives at a remote community in the NT [16]; the Audit and Best Practice in Chronic Disease (ABCD) project in the primary health care setting where certain ecological elements are incorporated into health promotion audit activities [17]; a recent literature review that discussed physical activity for First Peoples in a social ecological context [18]; and family wellbeing empowerment programs [19]. We have also identified such an approach in research reports of several other programs [20]. Given an increasing focus on the social and other environmental determinants of health in research and policy development for First Peoples in Australia, tools that provide a systematic way of evaluating the extent to which programs integrate an ecological approach are of potential value. This paper describes our experience of using one such tool in evaluating First Peoples’ health promotion programs and highlights the challenges and limitations in applying an ecological approach derived from a Eurocentric worldview in this context.

## Methods

### Research context in which an ecological approach was adopted for the evaluation of First Peoples’ health programs

Over the course of investigating community-based health program implementation with various community, non-government and academic organisations, several issues had arisen repeatedly in our experience. Firstly, routine reporting to program funders does not capture the depth, complexity and aims of First Peoples’ health initiatives; and secondly, there are few tools for systematically recording these aspects of health programs. These issues are also reflected in published research on First Peoples’ health, much of which has traditionally had a narrow biomedical focus [8, 20], and are not restricted to Australia [12]. The information presented here is effectively a case study of our attempts to use a tool designed for mainstream public health programs in Canada [4, 21] as a means to address these common issues. The ecological coding procedure was chosen because it has been applied in First Peoples communities previously [16, 22]. It is one of the few, if not only, tools that has the capacity to operationalise the extent to which programs are ecological and account for the social determinants of health in health programming.

#### The national research agenda of the Co-operative Research Centre for Aboriginal Health

From around 2002, the Co-operative Research Centre for Aboriginal Health (CRCAH) was establishing its *Chronic Conditions Program* of applied research at a national level. In part responding to outcomes of a health industry roundtable, the CRCAH’s agenda prioritised projects addressing chronic disease management approaches that involved families, organisations and communities [23]. The CRCAH 2007 publication *Beyond Bandaids* included a review of interventions that took a community development and empowerment approach to addressing the social determinants of health. The review took a broader view of what constitutes ‘social determinants’ than the Eurocentric definition in common use at the time, and identified a need for “methodologies capable of assessing and explaining community development and empowerment processes and outcomes” [24]. Like other work [12], the review noted the importance of a transdisciplinary approach to program design and evaluation and the need to involve industry partners to maximise the likelihood of research translation to practice.

The successor to the CRCAH, the Co-operative Research Centre for Aboriginal and Torres Strait Islander Health (subsequently renamed the Lowitja Institute) further developed this work though its *Healthy Communities and Settings Program* which took an overtly ecological approach to researching health program design and evaluation and had as its goal “An improved understanding of the determinants of Aboriginal and Torres Strait Islander health through the development and use of tools that more accurately measure enabling environments to improve the health and wellbeing of Australia’s First Peoples” [25]. The program included research investigating racism as a social determinant of First Peoples’ health and the implementation and evaluation of multi-level interventions in mainstream institutions to prevent racism [26, 27]. As an applied research program, expected outcomes included: better integration between addressing the clinical and social determinants of health; a framework, tools and capacity building for community organisations to monitor, evaluate and report on their programs, and develop health promotion services; meeting the challenge of attributing outcomes to programs in a context of multiple programs and stakeholders operating in community at any one time; and empowering communities to take a ‘bird’s eye view’ of program activities to inform local decision-making.

#### Developing a local research program to evaluate First Peoples’ health promotion

In parallel with the development of this national agenda, the development of a research program with ACCOs in northern Victoria over a number of years led us to try and address the barriers noted above to ongoing support for Aboriginal health promotion. Commencing in 2001 as a risk factor screening development program, *The Heart Health Project* evolved to also include investigation of determinants of wellbeing, development and evaluation of nutrition and physical activity programs for youth, and the establishment of a cross-organisational alliance for health promotion implementation and its evaluation using an ecological approach [9, 28–30]. It was also at this time that The University of Melbourne Department of Rural Health was being established in Shepparton in partnership with Rumbalara Football Netball Club, and the University started to engage with the community more broadly through the Koori Health Partnership Committee [31]. The *Creating Healthy Environments* project which evolved from these collaborations takes an overtly ecological approach to program evaluation as a means to a) better capture the design complexity of First Peoples’ health promotion, and b) develop feasible tools and processes to allow health promotion practitioners to better monitor, evaluate and report their activity. We hypothesised that a cross-organisational partnership would allow a more ecological – and thus more effective – approach to health promotion by drawing on the breadth of expertise and resources available to diverse organisations. The project was approved by The University of Melbourne’s Human Research Ethics Committee. The research process – oversight and decision making by a local Steering Committee representing the partner organisations, inclusion of community researchers as Co-Investigators, capacity exchange, privilege given to local interpretation of research data, co-authorship of research articles – reflect current ethical guidelines [32] and a rights-based approach to First Peoples’ health.

The collaborating community organisations have strong historical, cultural and social connections. They have been represented by community leaders on a project Steering Committee with The University of Melbourne from the outset. Building on earlier work in the region [31], a series of Memoranda of Understanding set out the principles of the working relationship between these organisations and the University, and explicitly place ultimate control of the research design, conduct and reporting in the hands of the participating ACCOs [28]. The Memorandum of Understanding is not a legally binding document however, the University has standard requirements relating to intellectual property which are acquisitive and obscured in legal jargon, and there are issues of First Peoples’ knowledge, intellectual property and culture that are not part of standard agreements. Hence some negotiation was required to meet ethical guidelines relating to First Peoples’ health research [32] and for consistency with principles of community control.

#### Working collaboratively

Implementing and evaluating an ecological approach to health promotion will most often require a cross-organisational, collaborative approach in order to effectively address the determinants of health. Working in partnership should, in theory, allow greater consistency with an ecological approach – the expertise, reach and resources of multiple organisations should allow more settings, targets and strategies to be incorporated into programs. However, particularly from a research perspective, there are clearly historical barriers for community collaborating with mainstream institutions. Issues of trust, capacity in both mainstream and ACCO sectors to engage, and the presence of resources and authority for working across institutions can all impact on the implementation of strategies for working in partnership. Community workers are subject to the pressures of time constraints, workload, organisational accountability, standard reporting requirements, local community issues, and other competing priorities. In addition, there remains a lack of appreciation for the aims and purpose of health promotion and its associated activities as implemented by First Peoples and organisations. Trust is as important when conducting community-based participatory action research with health promotion practitioners as it is in academic work that seeks to incorporate Indigenous knowledge. For the projects described here, our working groups consisted of University- and community-based researchers involved in Creating Healthy Environments, along with Aboriginal and Torres Strait Islander collaborators recruited for their specific local or regional expertise in health or health promotion. The long term working relationships between a majority of the working group members enabled a generally safe working environment to be established, in which diverse views could be presented and disagreement accommodated. In practice, project implementation relies on trust, which is only achieved through the development of good working relationships at a personal level between researchers and community representatives.

The discussion below is based on our experience of applying an ecological approach to evaluating these and other community-based health promotion activities in Victoria, and in a literature review and characterisation of First Peoples’ health programs that included environmental targets [20].

### A tool for monitoring program activity implementation

**Fig. 1:**
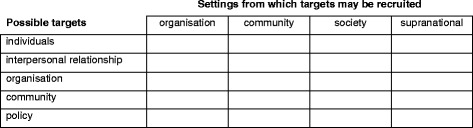
Grid for analysis of program activities according to the Ecological Coding Procedure (adapted from Richard et al. 1996) [21]

In 1996, Richard and colleagues published an analytical method for assessing the degree to which health promotion programs integrate an ecological approach. Based on the earlier work of McLeroy [1] and Simons-Morton [33], and drawing on systems theory [34–36], a method was developed that identified both the targets of a health promotion activity and the setting from which the participants are drawn [21]. It was designed to assess the extent to which ecological principles were integrated in health promotion programs in public health units in Canada [21, 37] by capturing information on specific activities implemented as part of a health promotion program. Richard’s bi-dimensional ecological approach builds on McLeroy’s single-dimensional ecological framework featuring intervention targets, the bidimensional typology underpinning Multilevel Approaches Toward Community Health [33] and the Precede/Proceed planning and evaluation model [38]. Thus interventions are coded on a grid according to two dimensions - the setting and the intervention target (Fig. 1). The higher categories of Miller’s *Living Systems Theory* [36] are used as settings for ecological analysis: organisations, communities,^1^ societies and ‘supranational systems’ (two or more countries). Settings refer to the places in which participants are reached or recruited into the activity and not the location where the activity is implemented. A health promotion program may reach or recruit participants in one or more of these settings. Within each setting, a number of targets are possible: the individual; the interpersonal environment; the organisation; the community; or political targets. These targets also can be networked to reflect the formation of community coalitions, collective governance structures, social support groups, mutual aid and other forms of social and family collectives.

According to Richard [21], an ecological program includes activities aimed at both environmental and individual targets and delivers these activities in a variety of settings. Richard, following Stokols [39], considers the degree to which a program integrates the ecological approach as a function of targets and settings: the more a program integrates individual and environmental targets across a range of settings, the more ecological it is. In addition, for a program to be ecological it must include a minimum of two intervention strategies: one with the client as a direct target and one strategy directed towards an environmental target. Finally, Richard’s ecological approach gives greater weight to the number of targets than to the number of settings in the program. The utility of this ecological coding procedure is that it distinguishes traditional health education strategies from health promotion efforts aimed at changing the broader policy and regulatory environment in order to improve community health and wellbeing. It is also a way of making sense of the different aspects of complex community health promotion programs.

The ecological coding procedure has been used by researchers from the Groupe de Recherche Interdisciplinaire en Santé from the Université de Montréal in their work with the Kanien’keháka (Mohawk) community of Kahnawake to monitor the Kahnawake Schools Diabetes Prevention Project (KDSPP) [22]. The procedure was also adapted for use in evaluation of First Peoples’ health promotion programs in the Northern Territory [16] and Victoria [30]. Domains incorporated in our version of the paper-based data collection instrument (used with permission from KDSPP) include: activity name, sponsoring organisation, date, description; whether it fulfilled its objectives on the day; target group, numbers of participants and where they were recruited from; health focus; role of local culture in activity design; barriers and facilitators to implementation of the activity.

## Results

### Data collection and analysis

As indicated, we have taken an ecological approach in several projects, including a review of published health programs (retrospective) and in an action research project describing and evaluating First Peoples’ health promotion (prospective). Both have involved close collaboration between university- and community-based researchers from diverse cultural backgrounds.

#### Retrospective use of the procedure as part of a literature review

The aim of our literature review was to identify Australian programs that had targeted one or more environmental determinants of First Peoples’ health. The rate of appearance of such reports has increased significantly over time [20]. After listing the specific activities that made up each of 24 relevant programs identified in research journals, we formed a working group to identify the target of each activity and categorise it using Richard’s ecological coding procedure. The target of an activity is defined as the entity (a person or a component of their physical or social environment) designated for change. Health promotion practitioners can seek to influence capacity development, knowledge, attitudes or skills of the persons designated for change. They can also seek to bring about changes in aspects of the ultimate target’s social and built environments or to strengthen relationships between community, interpersonal and organisational entities. Working from published articles without having contact with the practitioners who designed and implemented the programs necessarily involves a degree of interpretation, as we were relying on what information the authors (and journal editors) of the articles had available to them and chose to include. In practice, there were clear differences in interpretation of the target and intent of certain activities between mainstream and First Peoples’ perspectives.

Community-based researchers using a First Peoples’ perspective more frequently identified targets at the interpersonal, community and societal levels, reflecting the importance of social networks and relationships as outcomes, and the difficulty of distinguishing between family, organisations and community. In applying this method there is a risk of conflating the targets designated for change with the settings from where participants are recruited, thereby negating the value of Richard’s method, but coders were very clear that many activities, particularly those involving groups of community members, were primarily about strengthening interpersonal and community relationships. Treating ‘society’ as a target rather than as a setting only was a departure from the original method, which identifies only policy as a target in the societal setting [21]. Nevertheless, at this time in Australia’s history when there remain major unresolved issues of the status of First Peoples’ sovereignty and a high incidence of racial discrimination, ‘society’ (which we interpreted as meaning mainstream Australian society) could not be ignored as a legitimate target in this context. We did not seek to reconcile these different views, as we considered the difference an important outcome to report, with implications for how social determinants of health are defined and how interventions are funded, evaluated and “success” identified.

#### Prospective monitoring and evaluation of health promotion programs

The collection and analysis of prospective data using a collaborative approach allows a more accurate and precise analysis. In practice, researchers have generally completed the paper-based form by interviewing one or more health promotion practitioners about each specific activity associated with a given program, using a combination of open-ended and categorical questions about the nature and objectives of the activity. Specifying the aim, intended targets for change and the program setting of an activity sometimes required in-depth discussion with practitioners. Similar strategies were used in North East Arnhem Land [16]. In Victoria, we are moving towards having practitioners complete the forms in the interests of increasing practitioner ownership and sustainability of the process and the associated ecological approach. At present, identification of program targets, settings and strategies (the last describing the sequence of events linking the program to its targets) is conducted collaboratively between University- and community-based researchers. Greater involvement of practitioners in the analysis procedure is desirable. Our data collection tool in its present form may be overly complex for routine use in monitoring and reporting in ACCOs. However it is amenable to adaptation so that it focuses on the key elements of the ecological approach and addresses local organisational requirements, as Richard’s original article indicates [21].

Richard *et al*. (1996) also provided a framework in which to score programs according to the number of targets, settings and strategies (Table 1). To our knowledge the validity of this scoring system as a predictor of health outcomes or other aspects of program effectiveness, reach or sustainability has not been assessed. Nevertheless it provides an indicator of program complexity and changes over time and has been used as such [16]. Richard’s later review focussed on temporal trends in numbers of targets and the levels in which they were located rather than use this scoring system [4].

**Table 1:** Scoring method proposed for monitoring of an ecological approach to health promotion program design (from Richard *et al*. 1996) [35]

Characteristics of activities making up a program	Score
Only one intervention strategy, independent of the number of settings	0
At least two different intervention strategies, which did not include the direct targeting of the participants, regardless of the number of settings	1
One setting in which at least two strategies were implemented, one of which directly targeted the participants	2
Two settings in which at least two strategies were implemented, one of which directly targeted the participants	3
Three or more settings in which at least two strategies were implemented, one of which directly targeted the participants	4

## Discussion

### Data analysis

In our hands the ecological coding procedure has provided a useful way of collecting information about the complexity of Aboriginal health promotion, but only up to a certain point. The procedure was designed for health promotion programs delivered by government public health units, and is based on systems theories which are inherently Eurocentric (von Bertalanffy’s original, more inclusive philosophy notwithstanding) [36, 40]. It is therefore not surprising that we have encountered limitations in applying it to First Peoples’ health programs that arise from a more relational, collectivist approach. The development of an alternative systems framework that more accurately describes First Peoples’ relationships to family, community, Country and the broader society is currently underway, led by Aboriginal and Torres Strait Islander researchers (Marika *et al*. unpublished). This holistic framework is being used to culturally adapt Richard’s ecological coding procedure so it more accurately represents the collectivist and relational targets designated for change in health promotion programs. However, to date we have attempted to deal with these limitations by several means:

- Ignoring the blurred boundaries between levels in the First Peoples community context when identifying targets and settings – particularly between the interpersonal, organisational and community levels, where distinctions are often unclear regarding where roles of and within family groups/clans, organisations and broader community start and finish. This limits the precision of the method;
- Incorporating additional health foci based on First Peoples’ knowledge of health and its determinants in Victoria. Thus, in addition to physical activity, nutrition, weight loss and smoking as common health foci, information is sought about whether the activity focussed on history, sense of control, relationship with mainstream society, connectedness, or threats to wellbeing [9]. This has the advantage of systematically opening up the description of health promotion to more accurately reflect its aims and outcomes;
- In our earlier review, we argued that mainstream society and the individuals, relationships, organisations and communities of which it is comprised all have a strong influence on First Peoples’ health, and therefore are legitimate and necessary targets and settings if Australian health disparities are to improve. Strategies that reach people in mainstream settings are therefore especially important. To reflect this issue, and partly also for simplicity, we previously coded all targets outside of First Peoples’ communities as being within the level of ‘society’ [20]. This approach to coding is unsatisfactory because although it recognises the important and often overwhelming role of mainstream Australia as a determinant of First Peoples’ health, including through the enactment of interpersonal and structural racism, it fails to account for the possibility of a pluralistic society that can embrace diverse communities. As an alternative we have recently begun distinguishing ‘mainstream’ organisations and communities from First Peoples’ organisations and communities. This approach allows the identification and coding of “mainstream organisations (such as liquor outlets) that are the target of Aboriginal programs/activism” [20] while avoiding overly simplistic assumptions about the place of First Peoples in Australian society.

In practice, the extent of application of the ecological approach may be underestimated if a representative breadth of activity is not included in monitoring and evaluation. Cargo and colleagues made concerted efforts to include all possible health promotion activities of relevance by working across multiple organisations within the community, using a snowball approach, and by reference to published organisational reports [16]. The investigators noted that “Strong social networks between community members and researchers facilitated the identification of relevant activities”.

The Richard procedure is fundamentally focussed on the individual at the population level as the ultimate target of health promotion, and this is how the tool has been applied to date [16, 21, 22, 30]. This causes problems in coding activities from a First Peoples’ perspective, where in our experience family and community are sometimes identified as the ultimate target of a given activity. We also note that in the original report of the application of the procedure in a Canadian First Nations context there was not universal agreement regarding terminology used in coding, specifically the use of the term “political” to describe targets at the higher levels (societal and supranational) of Miller’s model [22], although the precise nature of this disagreement was not described.

### Working collaboratively

Like others, [4, 12, 16] we find a participatory and collaborative process the only means of obtaining relevant information about health programs in an ethical manner. We have previously noted that an important limitation of our review was a lack of local knowledge about most of the programs included [20]. Application of evaluation processes, regardless of the tools used, risks perpetuating the colonial project. Evaluation can only be implemented with community control of the process if accuracy, precision and human rights are to be achieved.

## Conclusions

Notwithstanding the limitations noted above, taking an ecological approach to health promotion and monitoring it using the ecological coding procedure developed by Richard [21] has value in terms of identifying the complexity and aims of First Peoples’ health promotion programs and how they seek to address the determinants of health at a local level and more broadly. Its application, if conducted by First Peoples, potentially aids the implementation of values and principles required for effective health promotion [3]. At a minimum, it expands the horizons for funding and evaluating effective programs and potentially provides an impetus for collaboration to address multiple determinants of health for community members. Underestimated evaluations of programs do not inform the stakeholder/funder exactly what is happening within that program, therefore the stakeholders are not able to recognise its true value in personal or economic terms. However further work is required to systematically evaluate the benefits, and risks, of this approach on a broader scale, and to interrogate the accuracy of existing systems theories as models for evaluating First Peoples’ health promotion.

If the ecological coding procedure or a modification of it is to become a transferable method across cultures and sectors, certain conditions will be required: that the ecological approach be valued as a step towards more accurate reporting and evaluation of health promotion; local community direction of the research and evaluation process; the theoretical basis of the method more accurately reflects First Peoples’ lived realities; clarity about the level of support, if any, required for data collection and analysis from the academic and/or government-level public health workforce; and its inclusion in the initial design of a program. Like us, other groups attempting to implement an ecological approach in partnership have identified barriers relating to cultural difference, staff turnover, adequate resourcing and accountability and reporting requirements of funders [16, 41]. Nevertheless, the knowledge of locally-relevant determinants of health and of program design, intent and outcomes can only come from the people involved in implementing those programs. Partly for this reason, working collaboratively to collect and analyse program information is an essential element of the ecological approach, both in our experience and internationally [4, 16, 42].

Thus the ecological approach in general, and Richard’s ecological coding procedure specifically, have potential to be used more broadly as a means to developing, describing and evaluating health promotion. However, certain refinements are required to the procedure in order to make it more accurately represent the aims and philosophy of First Peoples’ health promotion. In order to become generalisable and transferable, the ecological approach to health promotion and its evaluation needs to gain broader validation and recognition in the ACCO sector as an accurate reflection of their activity; greater understanding among program funders of the nature and purpose of Aboriginal health promotion, and of the resources required to enable a collaborative approach; and a commitment from policy makers to address the breadth of social determinants of health and to evaluate program activity accordingly.

## Abbreviations

ACCO: Aboriginal Community Controlled Organisation
CRCAH: Co-operative Research Centre for Aboriginal Health

## Footnotes

1 The ‘community’ level was added by Richard and colleagues in order to more accurately capture the targets of local-area health promotion activities.
